# Supplementary material for: Spectroscopic analysis reveals that soil phosphorus availability and plant allocation strategies impact feedstock quality of nutrient-limited switchgrass
Source: Commun Biol. 2022 Mar 11;5:227. doi: 10.1038/s42003-022-03157-7 (PMC8917137; doi:10.1038/s42003-022-03157-7)
Supplement: Supplementary file 2 — Supplementary Information [file 42003_2022_3157_MOESM2_ESM.pdf]

SUPPLEMENTARY FIGURES

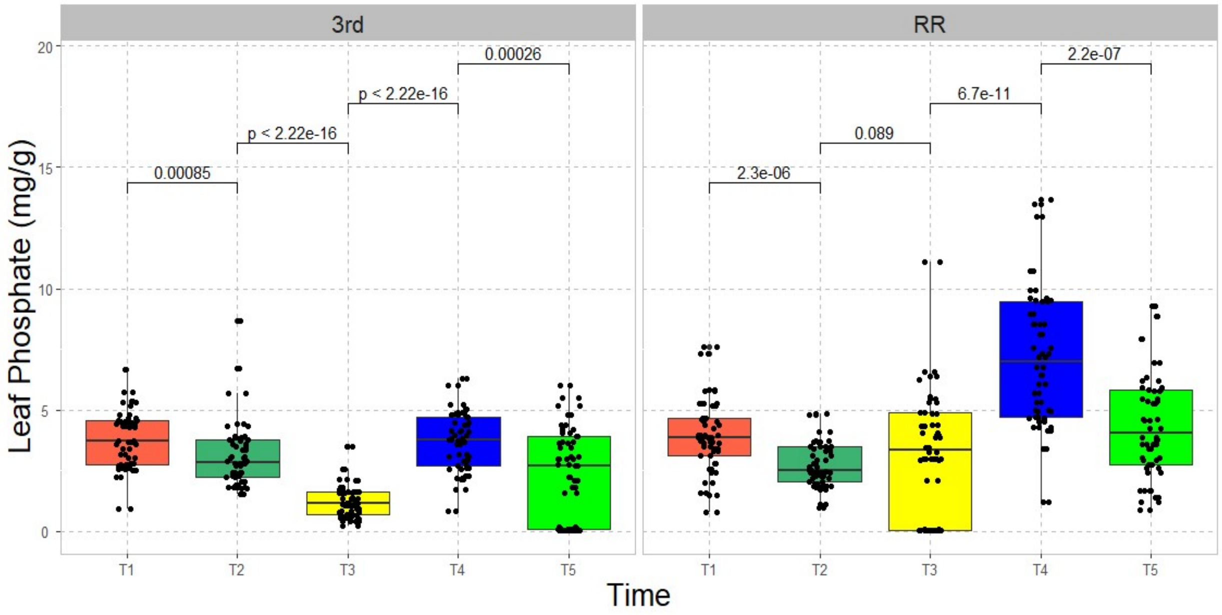

Figure S1. Seasonal changes of total P concentration in leaf tissue samples from plants grown in the two field plots, 3rd Street and Red River. *P-values* derived from pairwise comparisons are shown on the horizontal lines.

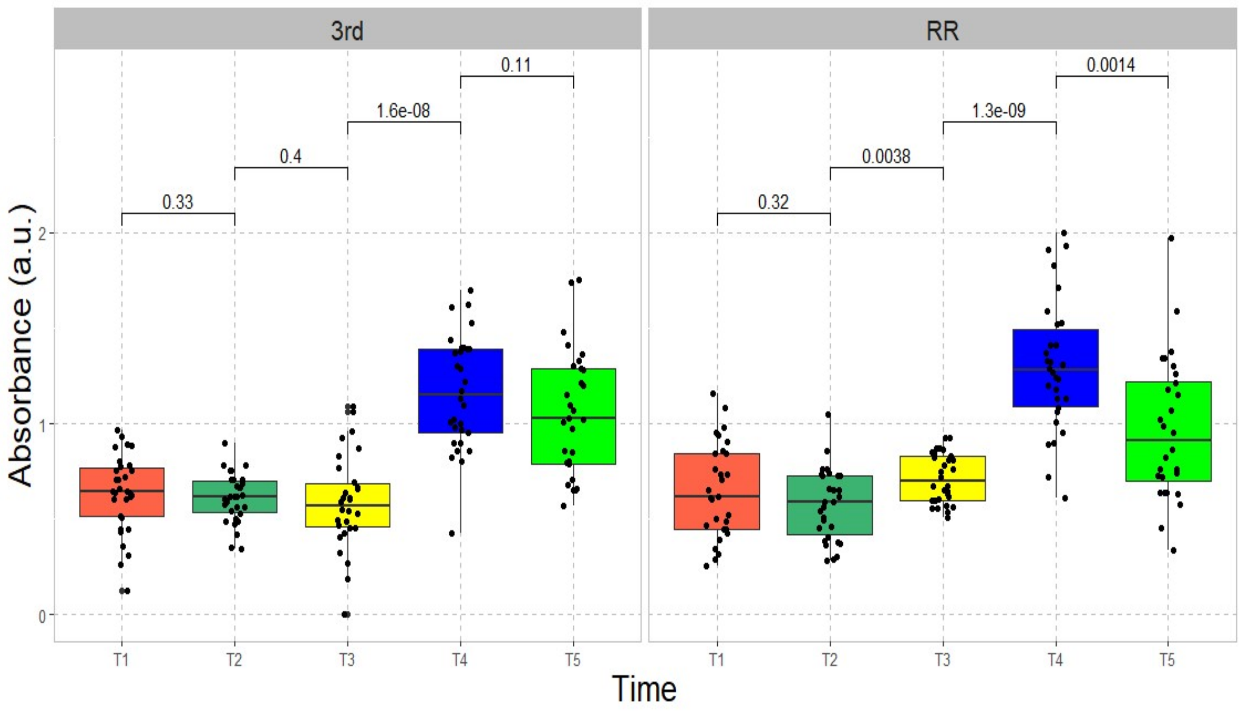

Figure S2. Seasonal changes of lipids in leaf tissue samples, from plants grown in the two field plots, 3rd street and Red River. *P-values* derived from pairwise comparisons are shown on the horizontal lines.

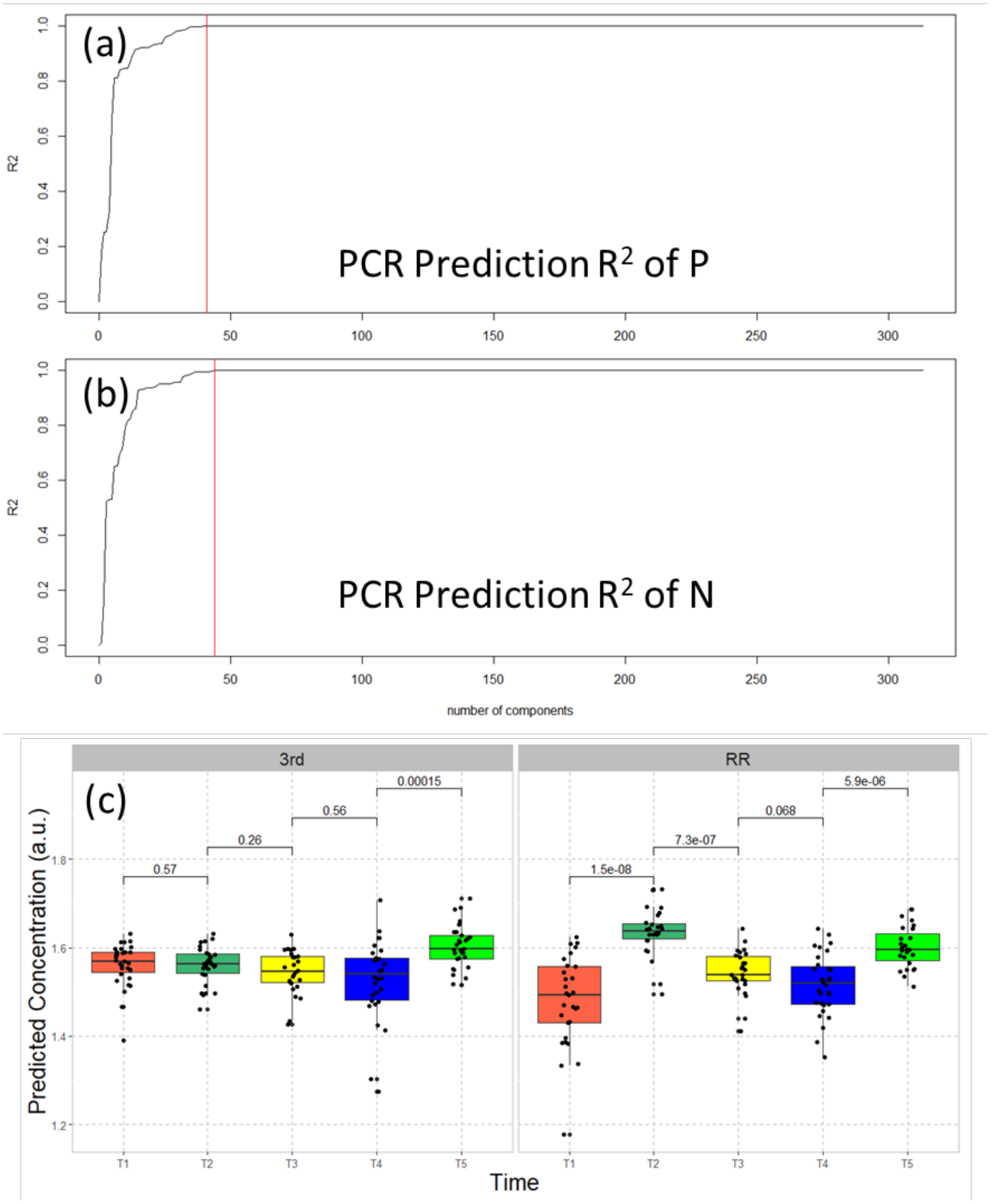

Figure S3. Learning curves (using a 10-fold cross validation) for PCR models showing 100% R^2 value with a model of 41 principal components in prediction of P concentration in the lab-based experiment (a), and 44 for N (b), and the predicted plant-available N concentration (c). *P-values* derived from pairwise comparisons are shown on the horizontal lines. Note that it's been shown

that although ion confounding effect between K^+ and PO_4^{3-} is not significant, it may be significant between K^+ and NO_3^-. [ref: Niedz, R. P., and Evens, T. J. (2006). A solution to the problem of ion confounding in experimental biology. Nat. Methods 3, 417–417. doi: 10.1038/nmeth0606-417]

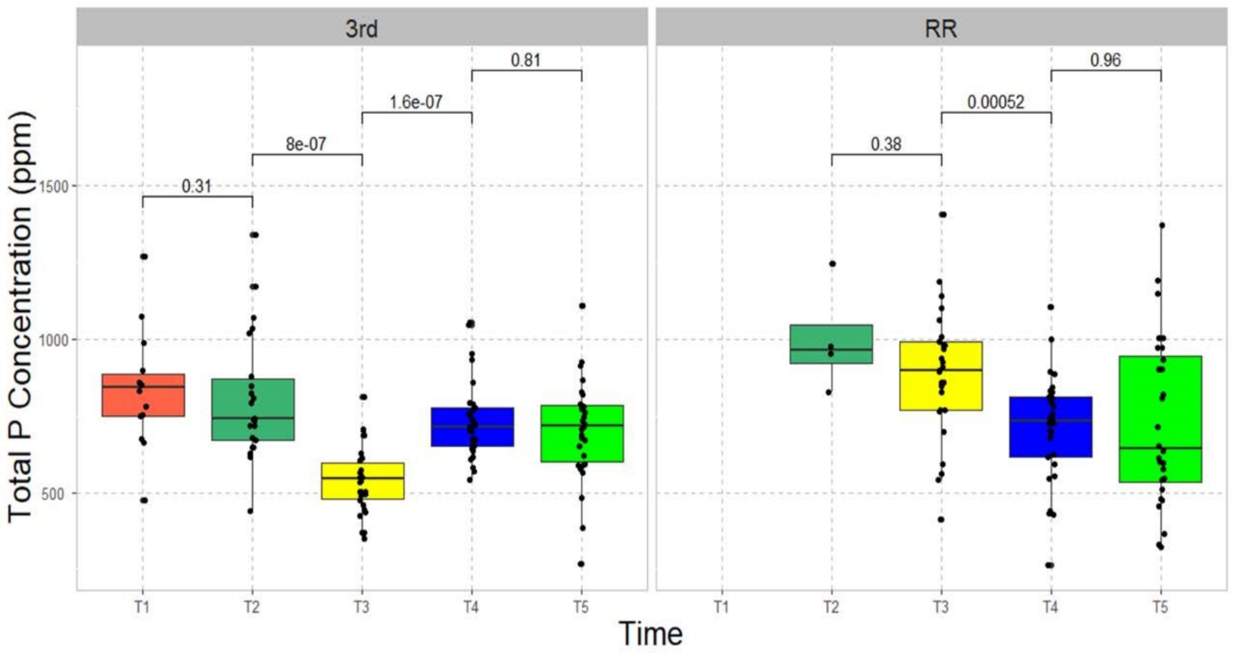

Figure S4. The seasonal dynamics of total P concentration in roots as determined by ICP-MS analysis of samples collected from field experiments, showing similar trends as of the predicted plant-available P concentrations in the rhizosphere. The concentrations at T5 are similar in the two plots (p = 0.6626), with a mean value of 729 ppm at 3rd St and 703 ppm at RR respectively. No root samples are available for the RR site at T1 because the roots were small. *P-values* derived from pairwise comparisons are shown on the horizontal lines.

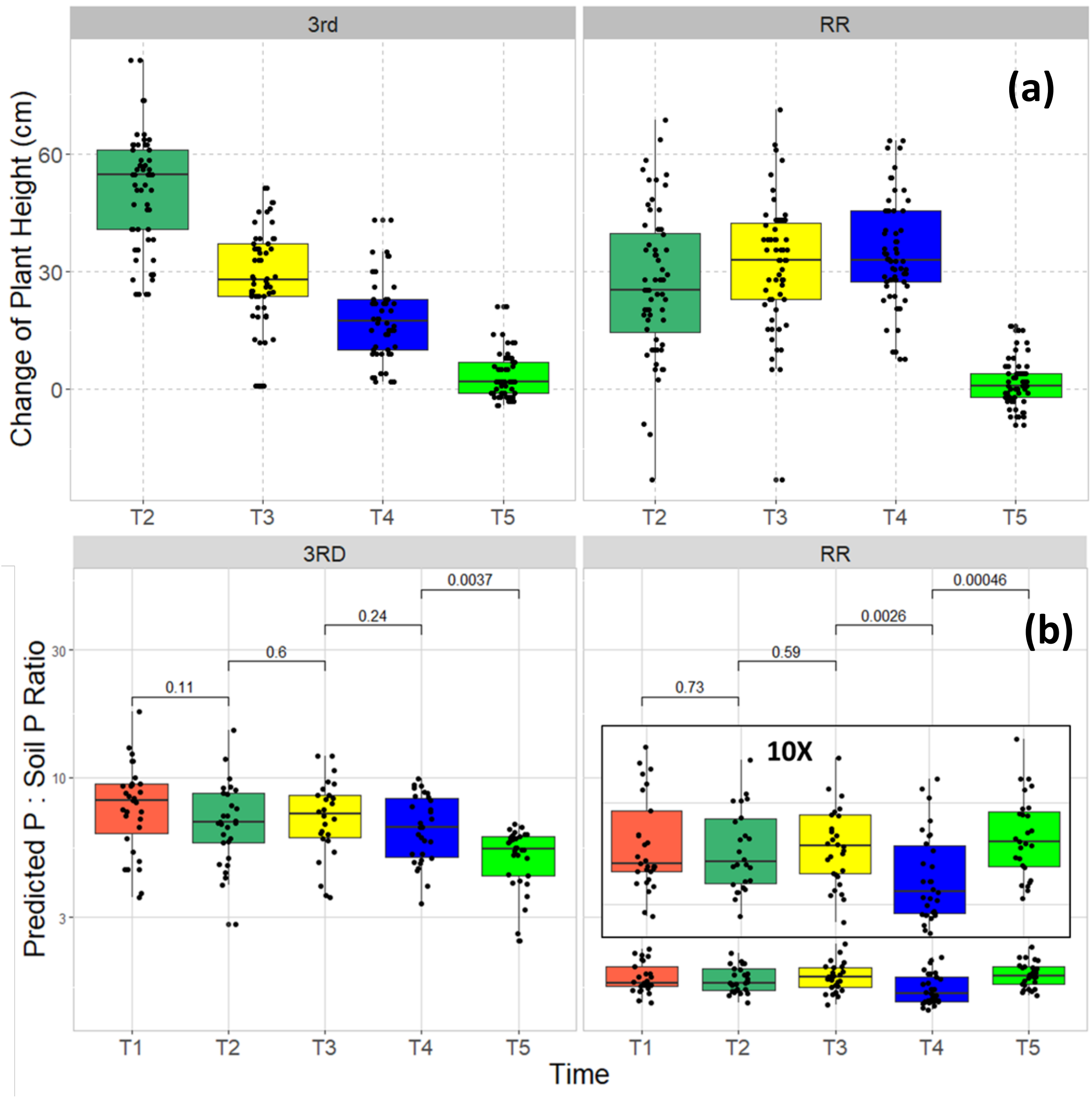

Figure S5. (a) Growth rate of plants at the two field plots shown here as the change of the plant height; (b) Seasonal change of the ratio between the predicted P concentration (Figure 4a) and the water-soluble P concentration in soil (Figure 2), with the inlet figure showing a 10 times zoom-in on the y-axis. *P-values* derived from pairwise comparisons are shown on the horizontal lines.

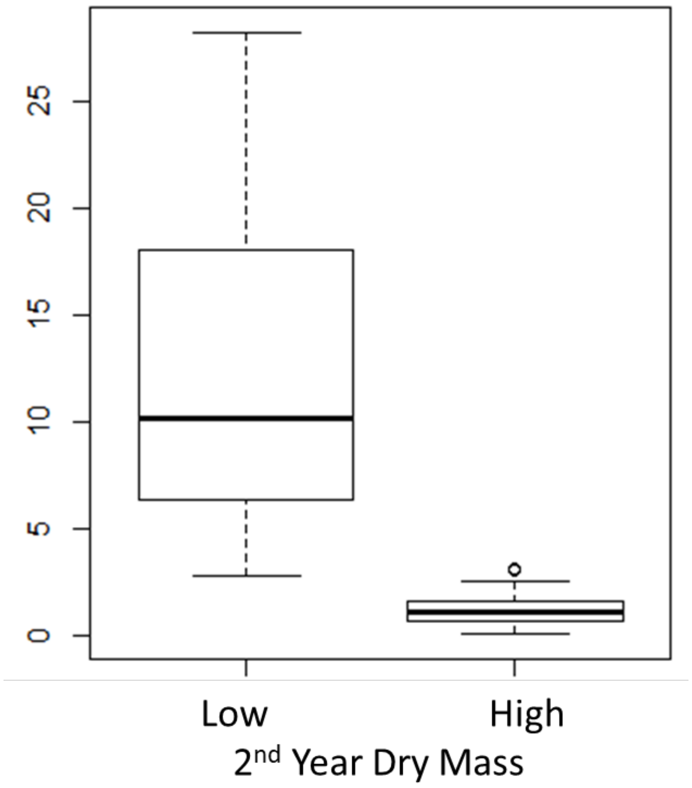

Figure S6. The strong association (p = 4.357 x 10^{-16}) between the 2nd year dry-mass performance and the P_i/P_o ratio in the late season.

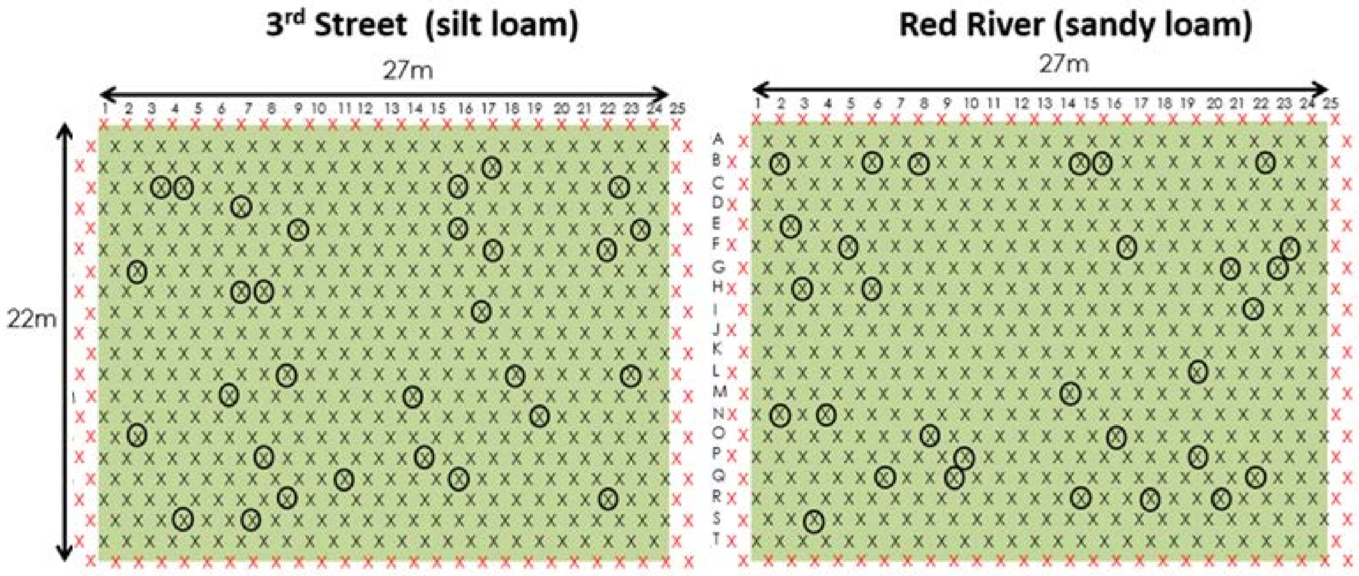

Figure S7. Plot design and sample selection in two field plots.

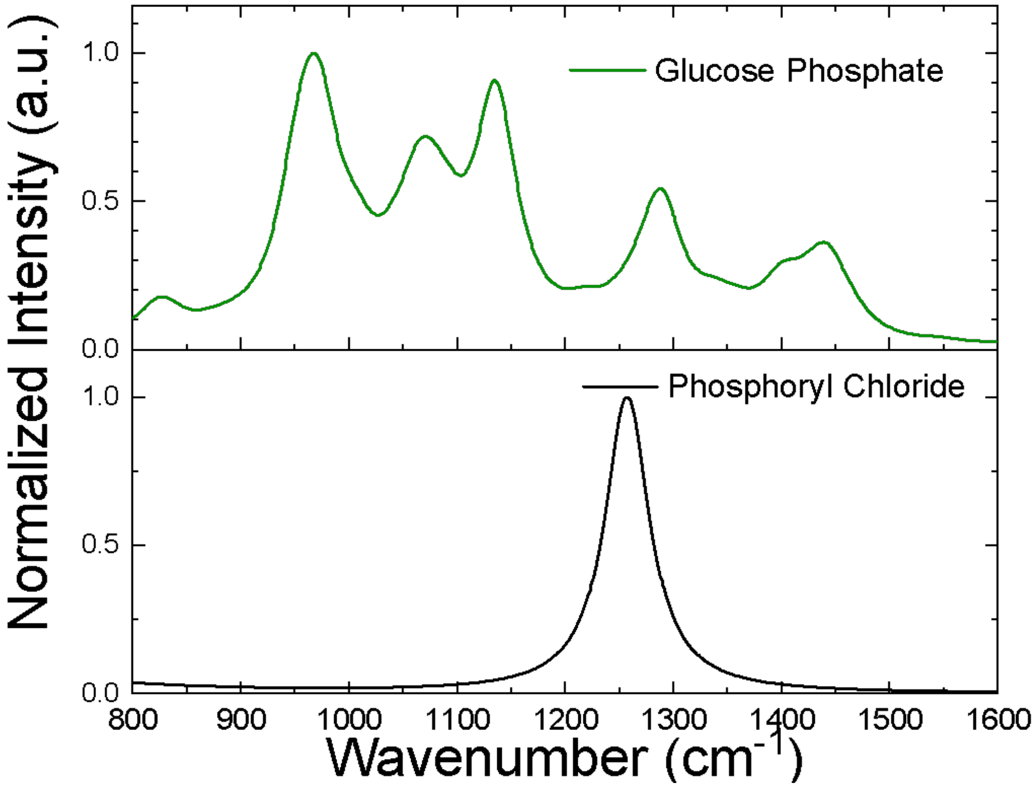

Figure S8. Quantum chemistry simulation of the infrared signatures of two model Pcompounds, glucose-6-phosphate and phosphoryl chloride. The Cartesian coordinates of the molecules were obtained from a PubChem database (National Center for Biotechnology Information, USA). The infrared spectra were reconstructed with a Lorentzian spectral linewidth of 25 cm^{-1} from an ab initio vibrational calculation using a hybrid density functional method (B3LYP/6-31G) [Y. Shao, et al., Phys. Chem. Chem. Phys., 8:0 3172, 2006].

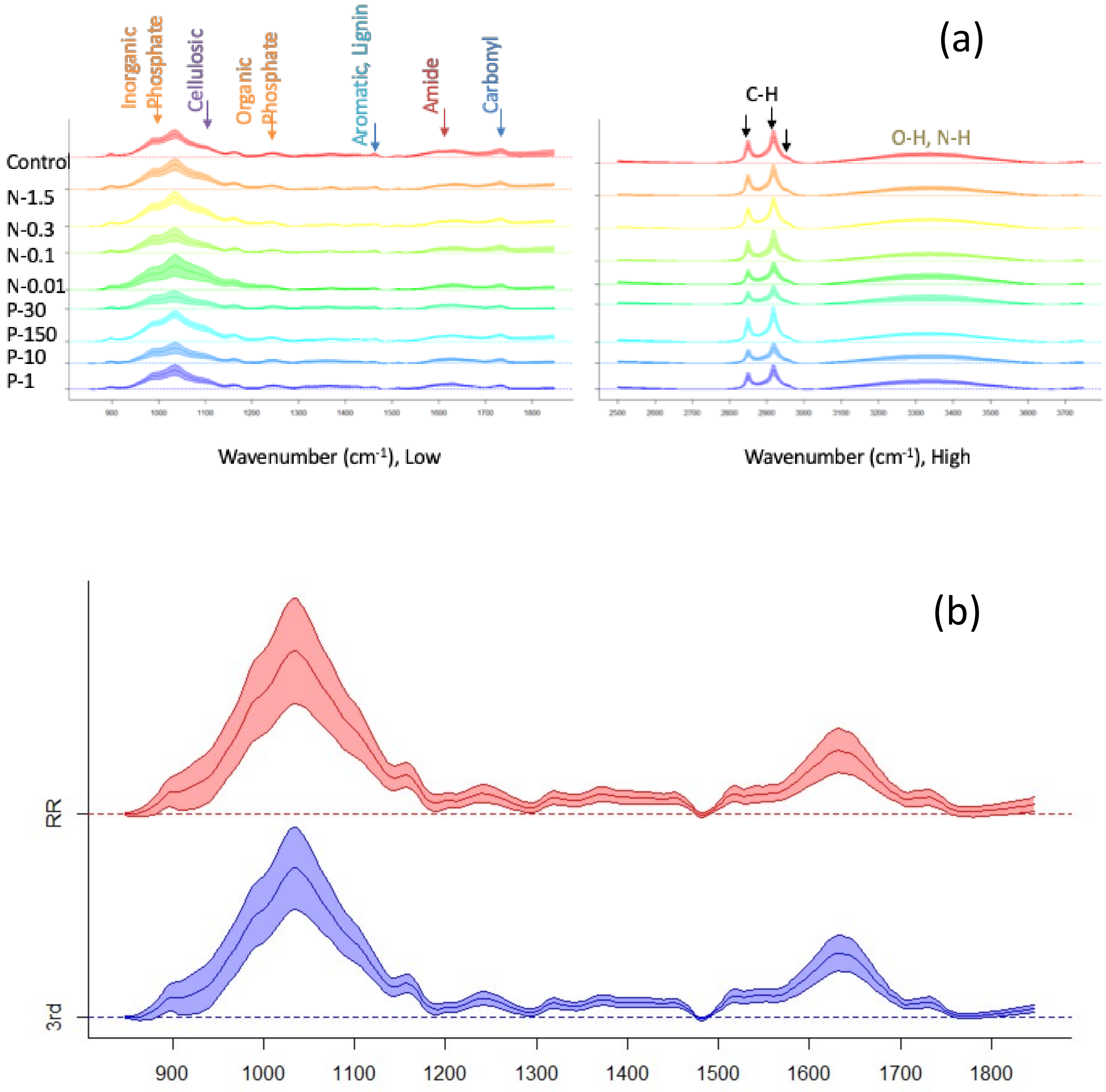

Figure S9. Averaged infrared spectra and their standard deviations in full frequency range for laboratory-based (a) and in lower half of the frequency range or “signature” region in fieldbased experiments (b), respectively. Peak locations of selected chemical groups are marked by the arrows in (a).
