# Supplementary material for: Spectroscopic analysis reveals that soil phosphorus availability and plant allocation strategies impact feedstock quality of nutrient-limited switchgrass
Source: Commun Biol. 2022 Mar 11;5:227. doi: 10.1038/s42003-022-03157-7 (PMC8917137; doi:10.1038/s42003-022-03157-7)
Supplement: Supplementary file 3 — Description of Additional Supplementary Files [file 42003_2022_3157_MOESM3_ESM.pdf]

Description of Additional Supplementary Files

File name: Supplementary Data 1
Description: The dataset includes tissue nutrient data from the lab-based experiments (Figures 1a and 1b), and tissue nutrient, soil P and model prediction data from the field experiments (Figures 2, 3 and 4).
