# Supplementary material for: Spectroscopic analysis reveals that soil phosphorus availability and plant allocation strategies impact feedstock quality of nutrient-limited switchgrass
Source: Commun Biol. 2022 Mar 11;5:227. doi: 10.1038/s42003-022-03157-7 (PMC8917137; doi:10.1038/s42003-022-03157-7)
Supplement: Supplementary file 5 — Reporting Summary [file 42003_2022_3157_MOESM5_ESM.pdf]

nature portfolio

Corresponding author(s): Zhao Hao, Eoin L. Brodie

Last updated by author(s): Sep 10, 2021

Reporting Summary

Nature Portfolio wishes to improve the reproducibility of the work that we publish. This form provides structure for consistency and transparency in reporting. For further information on Nature Portfolio policies, see our Editorial Policies and the Editorial Policy Checklist.

Statistics

For all statistical analyses, confirm that the following items are present in the figure legend, table legend, main text, or Methods section.

n/a	Confirmed	
☐	☒	The exact sample size (n) for each experimental group/condition, given as a discrete number and unit of measurement
☐	☒	A statement on whether measurements were taken from distinct samples or whether the same sample was measured repeatedly
☐	☒	The statistical test(s) used AND whether they are one- or two-sided<br>*Only common tests should be described solely by name; describe more complex techniques in the Methods section.*
☐	☒	A description of all covariates tested
☐	☒	A description of any assumptions or corrections, such as tests of normality and adjustment for multiple comparisons
☐	☒	A full description of the statistical parameters including central tendency (e.g. means) or other basic estimates (e.g. regression coefficient) AND variation (e.g. standard deviation) or associated estimates of uncertainty (e.g. confidence intervals)
☐	☒	For null hypothesis testing, the test statistic (e.g. F, t, r) with confidence intervals, effect sizes, degrees of freedom and P value noted<br>*Give P values as exact values whenever suitable.*
☒	☐	For Bayesian analysis, information on the choice of priors and Markov chain Monte Carlo settings
☒	☐	For hierarchical and complex designs, identification of the appropriate level for tests and full reporting of outcomes
☒	☐	Estimates of effect sizes (e.g. Cohen's d, Pearson's r), indicating how they were calculated

Our web collection on statistics for biologists contains articles on many of the points above.

Software and code

Policy information about availability of computer code

Data collection	We use a commercial software, Omnic, to collect infrared spectral data from the leaf samples.
Data analysis	We used statistical functions included in base package of R for the two-sample student t-tests and the multivariable analysis of variance tests, and add-on packages for spectral data processing (hyperSpec), PCoR (pls) and plotting (ggplot2 and ggpubr), with proper citations.

For manuscripts utilizing custom algorithms or software that are central to the research but not yet described in published literature, software must be made available to editors and reviewers. We strongly encourage code deposition in a community repository (e.g. GitHub). See the Nature Portfolio guidelines for submitting code & software for further information.

Data

Policy information about availability of data

All manuscripts must include a data availability statement. This statement should provide the following information, where applicable:

- Accession codes, unique identifiers, or web links for publicly available datasets
- A description of any restrictions on data availability
- For clinical datasets or third party data, please ensure that the statement adheres to our policy

All data are available upon request.

Field-specific reporting

Please select the one below that is the best fit for your research. If you are not sure, read the appropriate sections before making your selection.

☐ Life sciences ☐ Behavioural & social sciences ☒ Ecological, evolutionary & environmental sciences

For a reference copy of the document with all sections, see nature.com/documents/nr-reporting-summary-flat.pdf

Ecological, evolutionary & environmental sciences study design

All studies must disclose on these points even when the disclosure is negative.

Study description	We used a lab-based experiment to build our statistical model. In the lab experiment, the plants were watered daily to field capacity during a 4-week growth period with a nutrient solution containing 1, 10, 30, 150 or 500 µM of Pi, supplied as KH2PO4, and 0.01, 0.1, 0.3, 1.5 or 6 mM of N, supplied as KNO3. In the field, two plots with different P content were used in the study, and their different outputs were evaluated in this study.
Research sample	Two sets of samples were used in this study with the plants germinated from the same cultivar Alamo seeds. One was used in the lab study where plants were grown in a hydroponic system with daily watering with controlled nutrient solutions. In the field study, the plants were grown in two different field plots within the same region to reduce the impact of macro-scale climate variables.
Sampling strategy	All the 78 plants in the lab experiment were used in the study, while 60 plants in the field experiment, 30 per plot, were randomly selected from a total of 1000.
Data collection	The ICP-MS service was provided by Dr. Ivan Baxter at Donald Danforth Plant Science Center; The IC service was provided by David Huhman at Noble Research Institute. Dr. Zhao Hao collected infrared data from an ATR-FTIR spectrometer. All the detailed processes are included in the method section.
Timing and spatial scale	4 weeks of study on a total of 78 plants in the lab-based experiments. One year of study on a total of 60 plants in the field study.
Data exclusions	No
Reproducibility	A 10-fold cross-validation with random data partition were used in the machine learning process to evaluate the PCoR model accuracy. The rhizosphere prediction on the field data were validated with a second method (ICP-MS) with the corresponding root samples, and the FTIR derived organic P concentration was validated with a second method (IC) too.
Randomization	Field samples were randomly selected. In the model building we used bootstrapping to generate a dataset randomized with replacement for improved prediction accuracy.
Blinding	FTIR/ICP-MS/IC data were collected from independent researchers who didn't know/communicate each other with different set of sample labels (double blind).

Did the study involve field work? ☒ Yes ☐ No

Field work, collection and transport

Field conditions	“Third Street” with a silt loam textured soil and “Red River” (RR) with a sandy loam textured soil were used in this study. It's near the Oklahoma-Texas border, with a subtropical climate with average high/low temperatures of 34 C /22 C in summer and 12 C / -2 C in winter.
Location	These locations are referred to as “Third Street” (3rd St, latitude: 34.172100 N and longitude: -97.07953 W) with a silt loam textured soil and “Red River” (RR) near the Oklahoma-Texas border (latitude: 33.8820278 N and longitude: -97.2755056 W) with a sandy loam textured soil.
Access & import/export	The plants used are local.
Disturbance	The field sites were located in two research farms of the Noble Research Institute. Before planting, the plots were prepared with standard preparation procedure for farm plots, which includes tilling and application of pre-emergence herbicides. After planting, no herbicide, fertilizer or water was applied.

Reporting for specific materials, systems and methods

We require information from authors about some types of materials, experimental systems and methods used in many studies. Here, indicate whether each material, system or method listed is relevant to your study. If you are not sure if a list item applies to your research, read the appropriate section before selecting a response.

Materials & experimental systems

n/a	Involved in the study
☒	☐ Antibodies
☒	☐ Eukaryotic cell lines
☒	☐ Palaeontology and archaeology
☒	☐ Animals and other organisms
☒	☐ Human research participants
☒	☐ Clinical data
☒	☐ Dual use research of concern

Methods

n/a	Involved in the study
☒	☐ ChIP-seq
☒	☐ Flow cytometry
☒	☐ MRI-based neuroimaging
